# Spontaneous Isolated Superior Mesenteric Artery Dissection With Thrombosis: A Case Report of a Rare Presentation of Acute Abdominal Pain

**DOI:** 10.1016/j.cjco.2022.08.007

**Published:** 2022-08-19

**Authors:** Krishna Theja Reddy, Humera Syeda, Daniel Stenberg, Sina Bakhshaei, Srivaibhav Reddy, Mohammed Alkhero, Aminah Abdul Razzack, Sivaraman Gounder

**Affiliations:** a Cardiovascular Fellowship Program, UHS Southern California Medical Education Consortium, Temecula, California, USA; b Internal Medicine Residency Program, UHS Southern California Medical Education Consortium, Temecula, California, USA; c Internal Medicine Department, Karwar Institute of Medical Sciences, Karwar, India; d Internal Medicine Department, Dr N.T.R University of Health Sciences, Vijayawada, India

## Abstract

Spontaneous isolated superior mesenteric artery dissection is a very rare vascular disease that involves the superior mesenteric artery or its branches, with an incidence as low as 0.08%. The majority of cases occur in patients of Asian descent. Due to advances in imaging modalities, particularly abdominal computed tomography angiography, the diagnosis of this disease has been increasing. Herein, we present a rare case of spontaneous isolated superior mesenteric artery dissection with thrombosis in a young male patient with no past medical history. The importance of this disease as a differential diagnosis for acute abdominal pain is emphasized.

## Résumé

La dissection isolée spontanée de l’artère mésentérique supérieure est une maladie vasculaire très rare qui affecte l’artère mésentérique supérieure ou ses branches, et dont l’incidence s’élève à peine à 0,08 %. La majorité des cas surviennent chez des patients d’ascendance asiatique. En raison des progrès réalisés dans les modalités d’imagerie, et en particulier de l’angiographie abdominale par tomodensitométrie, le diagnostic de cette affection est en hausse. Nous présentons ici un rare cas de dissection isolée spontanée de l’artère mésentérique supérieure avec thrombose chez un jeune patient sans antécédents médicaux. L’importance de cette maladie dans le diagnostic différentiel de la douleur abdominale aiguë est mise en évidence.

Spontaneous isolated superior mesenteric artery dissection (SISMAD) is a rare vascular event, with an incidence ratio of 0.08%, that usually involves the superior mesenteric artery (SMA) or its proximal branches.^1^ Studies show that patients are typically in their fifth to sixth decade of life and are overwhelmingly male; the majority of cases appear to stem from Asia. Recently, the diagnosis of SISMAD has been increasing due to advances in imaging techniques and modalities, particularly abdominal computed tomography angiography (CTA). Various connective tissue diseases were suggested to play a role in its pathogenesis, but no specific underlying pathology has been identified in the literature to date.^1^^,^^2^ Common presenting symptoms with SISMAD are back pain, abdominal pain, and flank pain.

## Case

**Figure 1:**
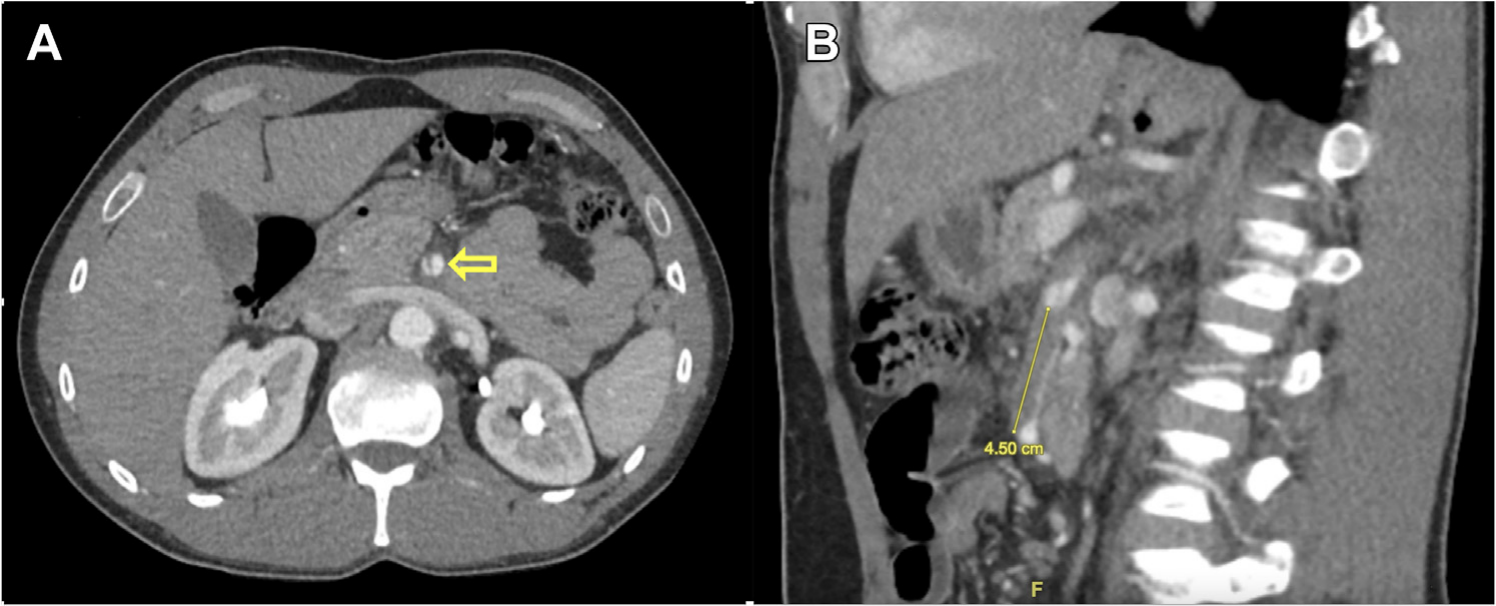
(**A**) Cross-sectional computed tomography angiography image of the abdomen shows proximal superior mesenteric artery dissection with an **arrow** indicating the intimal lining flap. (**B**) Coronal section of computed tomography angiography image of the abdomen shows superior mesenteric artery dissection with associated 1.3 x 4.5 cm thrombosis, causing 80% stenosis of the arterial lumen.

A 43-year-old Caucasian man, without known medical history, presented with sudden-onset abdominal pain in the epigastric area radiating to the back, associated with nausea and nonbilious vomitus. The remaining review of systems was negative. The patient denied history of abdominal surgeries, and the family history was negative for coagulopathy and connective tissue disorders. He also reported 20 pack-years of tobacco smoking but denied alcohol and substance abuse. Vitals were unremarkable on admission and throughout the course of the hospital stay. An abdominal exam was negative for rigidity, tenderness, and bruits. Laboratory measures, including troponin, serum lipase, lactic acid, lactate dehydrogenase, and a lipid panel, were monitored and remained unremarkable. Computed tomography of the abdomen with intravenous contrast revealed a low-density related proximal aspect of SMA, 2.5 cm from the origin of the artery, suspected for SMA dissection**.** Conservative management, including bowel rest, intravenous fluid therapy resuscitation, and pain management, was started. Due to the persistence of the symptoms, CTA of the abdomen was performed, which confirmed the dissection in the proximal part of the SMA (Fig. 1A), with associated 1.3 × 4.5 cm thrombosis, causing 80% stenosis of the arterial lumen (Fig. 1B).

**Figure 2:**
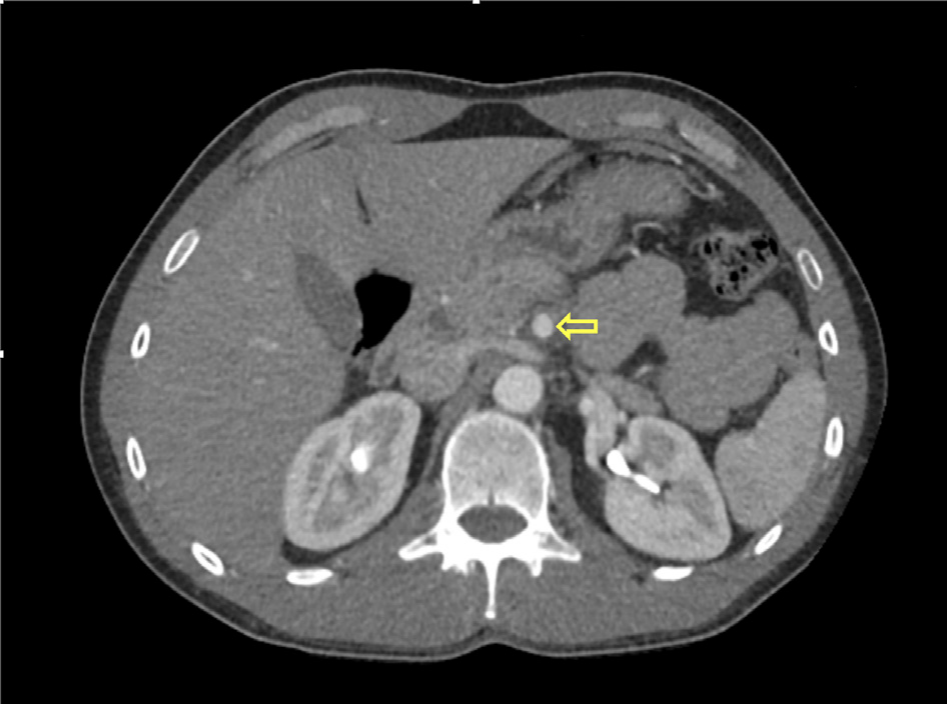
The 3-month follow-up computed tomography angiography image of the abdomen revealing remodeling of superior mesenteric artery and resolution of the dissection.

The patient was placed on intravenous heparin, and a vascular surgery consult was obtained. The intact flow to distal segments of the SMAs led to the recommendation of ongoing conservative management with anticoagulation. Serial lactic acid and lactate dehydrogenase levels were obtained to monitor for complications, including bowel ischemia, and remained unremarkable. After 48 hours of treatment, the patient’s symptoms resolved, and he was able to tolerate oral intake. The patient was discharged on apixaban, at 10 mg twice daily for 1 week, followed by 5 mg twice daily, for the duration of 3 months. A 3-month follow-up CTA of the abdomen revealed proper remodeling of the SMA and resolution of the dissection (Fig. 2). The patient has had a regular follow-up every 3 months for the duration of 1 year and has remained asymptomatic.

## Discussion

Vascular dissection occurs when a small tear in the intimal lining of a vessel exposes the deeper layers of an artery, and pressure forcibly separates these layers.^3^ SMA dissection can result from instrumentation or trauma, and rarely, it occurs as an isolated and spontaneous event.^4^ The extent of the dissection determines the nature and severity of the clinical manifestations.^5^ Several classification systems for SISMAD are used, with the choice generally based on the extent of dissection, the presence of thrombosis, and the patency of blood flow through the true and false lumen.^6^ Although no guidelines have been established for management, these classification systems can provide valuable information regarding the severity of the disease and assist with medical decision making. In general, if a patient presents signs of bowel ischemia or ruptured aneurysm, a surgical approach is required. Otherwise, conservative management (including antiplatelet or anticoagulant treatment, pain management, gastrointestinal rest, etc.) and close monitoring for bowel ischemia are recommended. CTA is recommended for persistent symptoms.^6^

Although conservative management is successful in many patients with SISMAD, operative or endovascular therapy is sometimes necessary despite the demonstration of flow in the distal vessels. Symptomatic patients treated conservatively are at risk of late secondary intervention, which occurs relatively often; therefore, long-term follow-up with imaging is warranted.^7^ The vessel is capable of remodeling over the first year, and follow-up computed tomography imaging may show resolution in approximately 10%-30% of patients. No specific duration is recommended for use of anticoagulants for SISMAD with thrombosis. If vessel remodeling is noted to occur over the interim, then the patient may be maintained on antiplatelet therapy alone for 1 year.^8^ In our patient, a 3-month follow-up abdominal CTA revealed remodeling and resolution of the dissection (Fig. 2); therefore, the decision was made to stop apixaban and keep the patient on aspirin alone for 1 year.

## Conclusion

According to the rarity of the condition, SISMAD often is not considered in the differential diagnosis for acute abdominal pain, out of proportion to the physical examination. CTA is a valuable imaging modality in these patients. Further studies are warranted to establish guidelines for the management and follow-up of SISMAD patients with thrombosis.

**Novel Teaching Points**

• SISMAD is a rare etiology of acute abdominal pain, with no established management guideline.
• Although a conservative approach with anticoagulants is successful in many patients with SISMAD and thrombosis, it occasionally requires operative or endovascular therapy.
• Follow-up imaging with abdominal CTA might be useful to assure artery remodeling.
